# Supplementary material for: Weight-normative messaging predominates on TikTok—A qualitative content analysis
Source: PLoS One. 2022 Nov 1;17(11):e0267997. doi: 10.1371/journal.pone.0267997 (PMC9624392; doi:10.1371/journal.pone.0267997)
Supplement: S1 Table — (DOCX) [file pone.0267997.s001.docx]

S1 Table. Codebook Definitions and Examples

| **Code:** | **Definition:** | **Example** |
| --- | --- | --- |
| **Content Perspective on Weight** | | |
| Weight inclusivity | Any content explicitly indicating that:  Anyone can achieve health regardless of weight  Everyone can engage in health behaviors regardless of weight  Not possible for everyone to achieve “normal” weight; there is a spectrum of weights just like there is a spectrum of heights | A creator dances in her room to music. The text on the screen reads:  “Just letting you know size will never determine happiness. Confidence is not size exclusive”  <https://uvmoffice.sharepoint.com/:v:/s/ResearchTeam222/EXUBQAC9OTRKuUKY4nXieuABGdCALJ8frSvq_D_iiNi5ww?e=O1x0TP> |
| Weight normativity | Any content explicitly indicating that:  Weight and health are cause and effect  There is a personal responsibility for weight and health, weight loss is promoted  Everyone can achieve a normal weight, and weight is the key to health | Video of a woman dancing and exercising. Her voiceover discusses her need to improve her health in the context of her weight:  “I have recently surpassed 200 lbs and I also found out from my doctor that I am extremely unhealthy.”  “I am passed borderline diabetic, I have PCOS and my blood pressure is out of control. I really need to make a change and lose a bunch of weight.”  <https://uvmoffice.sharepoint.com/:v:/s/ResearchTeam222/ESv4LpGcuuRNke_z9Tu8HwIBnOZzcgw2cCxsWH2egHUTKg?e=dYtVBg> |
| **Content of TikTok** | | |
| Nutrition advice | Creator provides nutrition guidance or beliefs | A dietician compares the nutrition advice she gives to the nutrition advice non-professional influencers give.  Example text:  “Me, a dietitian: ‘carbohydrates are the body’s most efficient source of energy. All foods can fit in a healthy diet.’ ”  <https://uvmoffice.sharepoint.com/:v:/s/ResearchTeam222/EaGcJLilKMhCg4WbeiBndP4BsHDF2qjiPqa_iI5cWCj-4w?e=FKgYQZ> |
| Food | Food is present in video  Subcode – Cooking: Creator provides or is cooking a recipe  Subcode – Takeout: Takeout food is featured or mentioned in video  Subcode – What I Eat in A Day: Creator presents what they are eating in a day | Video of a girl talking in front of the camera and peeling a clementine. Her plate is in front of her with pancakes, sausage, eggs, toast, and oatmeal.  <https://uvmoffice.sharepoint.com/:v:/s/ResearchTeam222/EdycEclP4YhLgU5xoIEq8sEBrI3lp6hZkAdedDM7ZStz_Q?e=lwH7DN> |
| Body image | Body image is discussed or portrayed in the video  Subcode - Body Image Positively portrayed by creator appreciating body features or displaying pride towards their body  Subcode - Body Image Negatively portrayed by creator expressing body dissatisfaction or a general sense of shame towards their body | Body image positivity:  Video of a woman in a bikini showing her body posed to look thinner and then unposed in a natural position. Captions are appreciative and loving of her body, noting that both views of her body are “fantastic” and “completely valid.”  “Please don’t let social media influence the way you see your body! U r perfect just the way u r.”  <https://uvmoffice.sharepoint.com/:v:/s/ResearchTeam222/ERr7PFeZZ4ZNgCZz3ArBc9oBBkO_yRg5uQQ5Rs9yuN5U_A?e=kD9wMS>  Body image negativity:  Video shows the creators body before and after quarantine wearing the same clothes to show her weight gain. The before video she is smiling at the camera, and in the after video she is sobbing and pulling at her shirt.  <https://uvmoffice.sharepoint.com/:v:/s/ResearchTeam222/EQc9O6TbKRZOkuylNtJ6Z7QBABSs1s5gEsHsOAVsT9ajVA?e=JxOo2d> |
| 3.4 Health | Video contains the hashtag health or the creator discusses a health behavior  Subcode - Physical Health or health behaviors  Subcode - Mental Health or health behaviors | A creator shows his body before and after a year. The voiceover explains his goals for the year and the health behaviors he planned to engage in, like “my goal for this year is to go on a diet, exercise every day and quit smoking  <https://uvmoffice.sharepoint.com/:v:/s/ResearchTeam222/EewuQXkFZYJPib9Yqjlm9n4BIcX27dqEvRQuWcwU25lXYw?e=xKFCl4>  The creator shows videos of herself doing physical activity but discusses her mental health in the text captions.  “I felt depressed. Thinking it was impossible.”  <https://uvmoffice.sharepoint.com/:v:/s/ResearchTeam222/EVu9SZDlEt1Dr_y4w51ldHEBn3R52zT_iG4V-lOuriyqFA?e=43PKQw> |
| 3.5 Diet | Portrays or references a diet such as keto, paleo, intermittent fasting, weight watchers, Atkins, etc. | Video of a young adult male explaining how he eats and exercises to build and maintain his physique. He shows a screenshot of his carb cycling template. In his voiceover he says:  “I have low carb days, high carb days, and one no carb a week.”  <https://uvmoffice.sharepoint.com/:v:/s/ResearchTeam222/EeL52n15NIFJqxX74liC_tkBx5MRLwqFLYpbY4LNEZHasg?e=0Cjuvx> |
| 3.6 Eating disorder promotion | Video glorifies an eating disorder or gives strategies for perpetuating the eating disorder | At the beginning of the video the creator poses in front of the camera in a cropped tank top. She posts a quote at the top of the screen, reading “omg ur stomach is so flat, what’s the diet?” She answers the question with images of gum, water, the oxygen symbol, ice cubes and tea, insinuating that she does not eat.  <https://uvmoffice.sharepoint.com/:v:/s/ResearchTeam222/EU4eCwowAyJAumjQD0c1ekEBvML9ChBz88O4rW4skxNtEA?e=kdXR0I> |
| 3.7 Eating disorder recovery | Video shows recovery steps or encourages recovery from an eating disorder | The creator shares everything that she eats in a day while recovering from an eating disorder.  “what I eat in a day (in eating disorder recovery)”  <https://uvmoffice.sharepoint.com/:v:/s/ResearchTeam222/Eei9-zKLbEVKiGpmNYeHrQ4B4GE8wGDh_5S7fYBBVbXCLw?e=MO3tLA> |
| 3.8 Health halo | A food is glorified for its health qualities such as salad or granola | Video shows black seed oil tablets and the creator stepping on a scaled. The voiceover attributes the tablets her weight loss. “For five days straight I started taking it and I lost ten pounds.”  <https://uvmoffice.sharepoint.com/:v:/s/ResearchTeam222/EYbb3ZvnDJVNkddxLlmDgt8BLnzg3okSs8E6fWXE5QPISg?e=bc5MuA> |
| 3.9 Calories | Creator mentions calories | Video of creator preparing her breakfast with music in the background. In the text captions, she mentions calories when discussing what she is making.  “I like to eat high calorie meals in the AM to keep me fueled”  <https://uvmoffice.sharepoint.com/:v:/s/ResearchTeam222/EaZobx0Jgy9PgV614ZZWa5sBxIFK42LPhoMmt2DPSchR0g?e=xuvlDe> |
| 3.10 Weight loss | Creator mentions weight loss  Subcode - Relationship related  Subcode - Quarantine weight loss | A man shows that the clothes he used to wear no longer fit him due to his weight loss. The text reads:  “Officially 11 months of successful weight loss!”  <https://uvmoffice.sharepoint.com/:v:/s/ResearchTeam222/EaEIxVSmMoNFgSvIQGvYV44BF2m-U-lA7mx1P6uywH0xhw?e=cGtShl>  Video begins with a picture of the creator and her boyfriend, and then switches to pictures of her working out. The text reads:  “Barely recognized myself in an unhealthy relationship a couple years ago….so I dropped him and 70 pounds.”  <https://uvmoffice.sharepoint.com/:v:/s/ResearchTeam222/EaDia4ZNJ8dCiVAMcvduzhQBr4wpMuDO7Tt30WdJmXrmcQ?e=ifdbzV>  Girl shows before and after pictures of her body, indicating weight loss, and shares her “quarantine ab workouts.”  <https://uvmoffice.sharepoint.com/:v:/s/ResearchTeam222/EU-_xVYQ6Y5Cij_zDGuLBC0Bs0zg9aZhrSAGXSniDswCSw?e=FwuMKU> |
| 3.11 Physical activity | Video portrays or mentions physical activity including dance | Video montage of the creator doing different workouts at the gym, like biking, jump roping, weightlifting.  <https://uvmoffice.sharepoint.com/:v:/s/ResearchTeam222/Eei9-zKLbEVKiGpmNYeHrQ4B4GE8wGDh_5S7fYBBVbXCLw?e=MO3tLA> |
| 3.12 Weight Bias/Fat Stigma | Subcode - Video explicitly combats weight bias/fat stigma by explaining the negative impacts of weight bias, or how to stop bias towards fat people and weight gain from occurring  Subcode - Video perpetuates weight bias/fat stigma by displaying dislike or assigning negative attributes towards fat people or gaining weight | <https://uvmoffice.sharepoint.com/:v:/s/ResearchTeam222/EY1Fop89QVJIhkWUI7WFd7IBZS-VTYkx84bc0wrGUV7PRg?e=3GcMc8>  Video of a teenage boy. The beginning shows quotes of negative things people have said to him about his body size, such as: “you would be hotter if you were skinnier,” and “he’s too fat for me.”  <https://uvmoffice.sharepoint.com/:v:/s/ResearchTeam222/EWb7Oc3XOghIp2j710U2eqQBzfgDldCafJbPok9uusCrxQ?e=PkIx8I> |
| 3.13 Culinary Instruction | Video teaches viewers how to make a recipe | Instructional video on how to make chicken teriyaki. Video shows steps and ingredients with voiceover instructions and captions.  “Preheat oven to 400. We’re going to be using skinless chicken tenders tonight. Place them in a large bowl. Add ½ cup teriyaki sauce.”  <https://uvmoffice.sharepoint.com/:v:/s/ResearchTeam222/EdpUvcRbbhBLuGcRBCy5VeMBVpppglsvqv88g3LEeUey1A?e=u7lJs9> |
