# Supplementary material for: Weight-normative messaging predominates on TikTok—A qualitative content analysis
Source: PLoS One. 2022 Nov 1;17(11):e0267997. doi: 10.1371/journal.pone.0267997 (PMC9624392; doi:10.1371/journal.pone.0267997)
Supplement: Attachment — Submitted filename: Reviewer Response_8_15_22.docx [file pone.0267997.s003.docx]

**Reviewer #1: This is an interesting study presented well however there are some concerns with regard to the contribution to scientific literature and rigor of methods.**

**1. The analysis done is a content analysis with quantification - please clarify this in the methods**

We have clarified that the analysis was a thematic analysis with quantification.

**2. There are some findings presented in the discussion such as - line 27 on page 5 An example pattern would be users showing their weight transformation, paired with explaining “what they ate on their journey.” and line 245 on page 13 Dialogue, sounding like a pep talk from a coach or a trainer, containing phrases such as “no excuses,” “get up” and “if you want it bad enough, you’ll do it,” implies that not trying to lose weight makes you lazy, and inferior to those who are pursuing weight loss. These findings have not been presented in the results..the analysis could include a thematic analysis -all transcripts (text) could be coded and then themes identified.**

We did conduct a thematic analysis, where we coded all videos including their dialogue, sounds, and actions to identify predominate themes. We have restructured our paper with a combined results/discussion divided by theme to more clearly delineate the themes we identified in the data.

**3. Much of the discussion reads meaning into the text of the videos this is not scientifically appropriate nor robust as a method**

We respectfully disagree that the discussion reads meaning into the text of the videos. The discussion section identifies themes that emerged from the videos and then uses examples from the videos to illustrate those themes. The discussion also explains why these themes may be helpful or hurtful to a viewer. The authors are not implying that a particular creator meant to for example, glorify weight loss, but any video that spoke positively of weight loss was coded into this theme, and then previous research has indicated how the glorification of weight loss may be harmful. In the discussion we hoped to help readers realize why the themes we identified were important, we meant to provide meaning to our themes, not to interpret particular videos beyond categorizing them into our themes. We have made sure to tone down any causative language that may have been in the discussion which hopefully will help with this concern. If you can provide specific examples of where you feel we have over-reached in our discussion, we are happy to revise.

**4. Limitation - please mention that this reflects only one social media platform and the study would have been richer if other platforms such as Instagram would have been included**

We have added this as a limitation.

**5.Please specify clearly what time period the data was collected- some places 'fall' is mentioned and another place September...this analysis is very sensitive to time and what was trending then may not be trending now..please mention in limitations**

The posts were collected in September 2020, we have specified this throughout the manuscript. We have added a limitation about how trends continue to change on TikTok, although the hashtags we analyzed have substantially more views today than they did when we analyzed them, indicating that they continue to be popular on TikTok.

**6. The discussion currently presents new data with interpretation..please consider deepening the analysis on what the study tells us, who it would benefit what kinds of action can be recommended ..how this study contributes or relates to other scientific literature.**

We have restructured the paper to have a combined results/discussion section presented by theme. We feel that this structure better presents our quantitative and qualitative data. In the discussion section we have also added several tie ins to previous literature on social media. We have recommended that adults help young adults curate their social media feeds and improve their evidence analysis skills. We also think it’s important for experts to begin to engage on social media platforms to highlight weight-inclusive content. We have highlighted these suggestions in our discussion. As this paper didn’t study possible solutions to the weight-normative inaccurate content on TikTok, we can only offer suggestions for future research.

**Editor’s Comments**

We believe we have addressed all of the editor’s comments. Thank you.
