# Supplementary material for: Weight-normative messaging predominates on TikTok—A qualitative content analysis
Source: PLoS One. 2022 Nov 1;17(11):e0267997. doi: 10.1371/journal.pone.0267997 (PMC9624392; doi:10.1371/journal.pone.0267997)
Supplement: Attachment — Submitted filename: PLOS ONE Response To Reviewers 9_26_22.docx [file pone.0267997.s006.docx]

**PLOS ONE Response To Reviewers 9/26/22**

**Journal Requirements:

Please review your reference list to ensure that it is complete and correct. If you have cited papers that have been retracted, please include the rationale for doing so in the manuscript text, or remove these references and replace them with relevant current references. Any changes to the reference list should be mentioned in the rebuttal letter that accompanies your revised manuscript. If you need to cite a retracted article, indicate the article’s retracted status in the References list and also include a citation and full reference for the retraction notice.**

We have checked all references and do not believe any articles have been retracted. We also updated the reference formats for several of the references to make sure they had correct links included. We are happy to update additional references if anything has been missed. Thank you!

Editor’s Comments

**Lines 243-246. Not sure you can make this interpretation. I believe you could say that those sentences imply that one might be lazy or lack strong will to move or do something, bu I cannot see how it can directly imply inferiority and social comparison. Please adjust sentence. Also, find some references that support this interpretation of yours.**

We agree that perhaps the dialogue does not imply social comparison directly. We have revised and removed the social comparison language, replacing it with an interpretation that the dialogue implies that not being able to lose weight is a personal failing of motivation. We hope this edit addresses the editor’s concern.

**Adjust sentence. There are two "reported". Sentence is confuse.**

Thank you, the sentence has been revised.
